# Indigenous Men Adhering to Highly Active Antiretroviral Therapy: Navigating Through Culturally Unsafe Spaces While Caring for Their Health

**DOI:** 10.3389/fpubh.2020.569733

**Published:** 2020-09-22

**Authors:** Meck Chongo, Josée G. Lavoie, Javier Mignone, Nadine R. Caron, Henry G. Harder, Rob Chase

**Affiliations:** ^1^Faculty of Medicine, School of Population and Public Health, University of British Columbia, Vancouver, BC, Canada; ^2^Center for Excellence in Indigenous Health (CEIH), School of Population and Public Health, University of British Columbia, Vancouver, BC, Canada; ^3^Department of Community Health Sciences, Rady Faculty of Health Sciences, University of Manitoba, Winnipeg, MB, Canada; ^4^Rady Faculty of Health Sciences, Indigenous Institute of Health and Healing, University of Manitoba, Winnipeg, MB, Canada; ^5^Northern Medical Program, Dr. Donald Rix Northern Health Sciences Center, University of Northern British Columbia Canada, Prince George, BC, Canada; ^6^School of Health Sciences, University of Northern British Columbia Canada, Prince George, BC, Canada

**Keywords:** indigenous, historic trauma, survivor, adherence, resilience, post-traumatic growth (PTG)

## Abstract

**Introduction:** Indigenous peoples in Canada have endured and continue to experience the impact of colonization by European settlers. The deleterious manifestations of intergenerational historic trauma (HT) are evidenced in the high HIV/AIDS epidemic-related premature mortality rates among Indigenous men, despite the availability of novel highly active antiretroviral therapies (HAARTs).

**Aim:** The aims of this study were to explore the impact of historic trauma (HT) on treatment adherence and health promoting practices among Indigenous men living with HIV, and how resilience was both expressed and mediated by survivor status.

**Methods:** This interpretive description study incorporated a cultural safety lens. Through partnership with the Vancouver Native Health Society, 36 male HT survivors were recruited using purposive and theoretical sampling. They told their lived experiences and health promoting practices with respect to HAART adherence through interviews and a focus group.

**Results:** Two broad categories (findings) emerged: (1) resilience as facilitator of HAART adherence; and (2) differential views on HT's impact. Resilience was expressed through nine concepts.

**Conclusion:** Most Indigenous men in this study demonstrate health promoting behavior, stay on HAART and have better health and well-being even if the environments they live in are marginalized or heavily stigmatizing. This study shows that areas of strength and adaptation, including factors promoting resilience can be harnessed to foster HAART adherence. With a consideration of these areas of strength and adaptation, this study offers implications for research and recommendations to improve treatment-adherent behavior, fostering healing from HT, and reducing HIV/AIDS-related deaths.

## Introduction

Indigenous peoples in Canada have endured and continue to experience the impact of colonization by European settlers. Through coordinated marginalization, forced dispossession, conquest and subjugation, settlers sought to eliminate Indigenous peoples as distinct nations and assimilate them into the Euro-Canadian mainstream (1). Apart from forced land dispossession, Canada's establishment of the government-funded, church-administered residential school system (from 1857 to 1996), the subsequent over-representation of Indigenous children in the child welfare system (2), coupled with persistent socioeconomic disadvantages, have left in their wake pernicious and egregious ripple effects of “historic trauma” that impact the capacity of Indigenous peoples for self-sustenance and maintenance of good health and well-being across generations (3, 4). Historic trauma (HT) is a cluster of traumatic events that operate as a causal factor in a variety of maladaptive social and behavioral patterns; or, it is a cumulative damage to emotional and psychological being across generations secondary to enormous tragedies. Hidden collective memories of trauma, or a non-remembering thereof, are passed from generation to generation (5).

The deleterious manifestations of intergenerational HT are evidenced in the high HIV/AIDS epidemic-related morbidity and premature mortality rates in Indigenous peoples in general, and among Indigenous men more specifically, despite the availability of novel highly active antiretroviral therapies (HAARTs) (6–9). In 2016 in Canada, the estimated HIV prevalence rate among Indigenous peoples was twice that of the general population. Indigenous peoples made up 11.3% of all new HIV infections while accounting for only 4.9% of the total population in the same year (10). In 2018, of the reported cases, 70.7% of newly diagnosed HIV cases in Canada were male, with 19.3% of them being Indigenous (11). Taking into account possible overestimation error, the reported average HAART adherence in British Columbia is 71% in men in general and 54% in Indigenous men in their 1st year of therapy (12, 13). This indicates that HIV-positive Indigenous persons do not have optimal access to HAART overall (14). Despite the challenges posed by intergenerational HT and ongoing disadvantage in socioeconomic, environmental, and structural provisions, many culturally grounded Indigenous men living with HIV remain strong and true to their traditional heritage. Some Indigenous men have found ways to deal with their trauma (15, 16), and report that HT does not greatly affect their adherence to treatment (17). Adherence is defined as taking above 80% of doses of prescribed HAART regimen as measured over a 24-weeks period of treatment, depending on patient and medication specifics (pharmacokinetics and pharmacodynamics) as newer, combination formulas are generally easier to adhere to (18, 19).

This study explored the impact of HT on treatment adherence and health promoting practices among Indigenous men living with HIV in Vancouver, British Columbia, Canada. The study investigated the experiential knowledge of Indigenous men living with HIV, focusing on the steps that survivors take in developing health promoting practices and adhering to HAART despite continued experience of HT and unequal social determinants of health. This manuscript emerges from the primary author's doctoral dissertation, “Experience talks, resilience shapes” (20), and is intended for knowledge translation, helping inform policy and decision-making for remedying HIV/AIDS' long-term effects across generations. Indigenous peoples continuously impacted through various means by HT are denoted here as “survivors.” “Indirect survivors” is used to identify Indigenous peoples whose HT experience is not via direct experience of HT, for example through coercion into residential schools or foster homes, but rather through intergenerational transfer, vicarious or other secondary means.

## Resilience in Indigenous Peoples – A Misconstrued Yet Vital Living Concept

Most survivors used the term “resilient” or “resilience” in describing their health promoting behaviors. Although a consensus on the definition of resilience has yet to emerge, what follows is an attempt to explain its use in the literature. Dulin et al. have argued that in earlier resilience research, the concept was framed as either a trait or unique capacity, some form of mental immunity, or the elastic ability to spring back to pre-crisis levels following exposure to single traumatic events (21, 22). All these notions are short in critical reflection and conscientization (a continuing process of awareness of causal conditions or factors and effects of oppression and subsequent mitigating action) and do little to foster acquisition of power, knowledge and skills to enable change (23–25). The old notions taken individually negate the history of cultural genocide and larger ongoing socio-economic and environmental factors promoting intergenerational trauma (26, 27). Most health researchers now regard resilience as a dynamic developmental process through which an individual or group overcomes past or current experience of adversity/trauma and its negative consequences, positively copes or adapts and is able to utilize sustainable resources for health and well-being (28–30).

Noteworthy, Indigenous peoples have lived according to their own set of values adequately meeting their needs, sustaining them and helping maintain their health as individuals and communities prior to contact (31). For most Indigenous peoples in Canada, resilience is not just overcoming trauma but also a philosophy of inevitable individual and communal cultural survival and persistence attributed to faith in the ancestors, families, and traditional teachings (32). Resilience is grounded in ordinary positive experiences (through friendships and family support) reflected in Indigenous peoples' spiritually-based natural health and strengths-based principles, maintenance of hope and a strong will (33).

During colonization Indigenous peoples endured trauma and were deprived of their tools of resilience, the expression of their identity through language, beliefs, rituals, ceremonies and cultural practices, Indigenous institutions and land (34, 35). Resilience is thus a long non-figurative process of healing that lies within one's Indigeneity. It should mean Indigenous peoples' conscientization, self-determination, and reclamation of tenets of identity, their past, present and future (36, 37). This is a challenge however, as to date Indigenous self-determination continues to be undermined as the balance of power privileges non-Indigenous counterparts.

With respect to resilience, research has shown that its development may be mediated by a form of “steeling effect,” which posits that resilience increases with exponential exposure to trauma (38). This notion is however misconstrued and romanticized by some who push the narrative that such exposure to cycles of trauma emboldens one's conviction to become more resilient and survive (39). It remains imperative to recognize that Indigenous peoples are not inherently vulnerable. As such, like in the general population, not all who experience trauma develop psychopathology or impaired health, but trauma has always been and remains immensely detrimental. Further, ascribing a positive connotation to Indigenous peoples' horrendous experiences of HT blunts the reality about the system's continued negative influence on Indigenous identity. We therefore need to be wary of loosely flouting impertinent and immaterial resilience narratives not considered with such reflection as they risk damaging healthy individuals and communities.

## Methods

This interpretive descriptive (40, 41) study was conducted in the British Columbia city of Vancouver with 36 Indigenous men, between the ages of 15 to 64 who were living with HIV and receiving HAART. The philosophical underpinnings of ID are that reality is subjective, complex and contingent on social context, and that knowledge is historically and culturally located but partly objective. Knowledge was thus generated through researcher-participant interactions involving unstructured interviews, reflective techniques that included memoing (42), and critical examination, woven together to generate deeper understandings.

Development of the research purpose and early design decisions were informed by extensive informal discussions with Indigenous men living with HIV at events relevant to HT and HIV/AIDS at the Vancouver Native Health Society (VNHS) where the lead author volunteered for seven years. Being non-Indigenous, the lead author found his place and continued to grow, recognizing that there are many ways of knowing and that harmony can be attained between these, and also that in recognizing and acknowledging this, personal growth can be attained leading to a better life for all. Delving into Indigenous research, locating oneself as a researcher was important (43, 44) as this enabled for identification of potential preconceptions and biases from own lived experiences of colonial subjugation, previous professional work experience in medicine, public health and research, setting these aside making sure these do not interfere with the study overall. Locating oneself also enabled for reaching into deeper levels of reflective journaling throughout the process and allowed for people to know the researcher. Further interactions with these Indigenous men helped build relationships of trust that enabled survivors to decide freely whether to take part in the study. In many areas, including that of HIV research and services access, the gap persists between Indigenous peoples, who are at a disadvantage, and the general Canadian population (45). Working toward shortening the equity gap in a culturally safe manner while building on Indigenous resilience is therefore vital for improvement of the health and well-being of Indigenous communities in the long term.

Ethics approval was obtained from the Research Ethics Board at the University of Northern BC and the VNHS Research Committee. Data was collected by the principal investigator, between August 2015 – July 2016, through one-to-one interviews with 27 participants and a focus group discussion with 9 other participants using open-ended questions. All participants were recruited through purposive sampling (46) and theoretical sampling (47) to either build on underdeveloped areas or clarify particular abstractions/issues. Participants were informed verbally about the study during men's events in the community and that steps would be taken to ensure confidentiality. Informed consent was given by survivors who agreed to take part.

The focus group and interviews were digitally recorded, transcribed verbatim by a professional transcriptionist, coded and analyzed using NVivo 10 qualitative data analysis software. Analysis conducted by the lead author involved creating broad concepts grounded in the data using line-by-line coding followed by a framework matrix (48), focusing on specific issues highlighted. Each cell reflected coded quotes related to the intersecting row (survivor – denoted by letter and number) and column (concept). Concurrent collection of data and constant comparative, iterative analysis was employed allowing for identification of similarities and differences, and continually refining concepts and relevant theoretical categories allowing for emergence of theory from the data. Further analysis compared individual participant's distinctive perspectives according to survivor status. Collaborating online, categories to use in organizing concepts were agreed upon by co-authors.

## Findings

Key demographic information collected from the 36 survivors is shown in Table 1. Of the participants recruited, four (11.1%) were ≤ 24 years old, and nine (25.0%) were indirect survivors. The age range was from 21 to 64 years (average 44 years). The time lag from first diagnosis to therapy ranged from 0 to 17 years: 26 survivors began therapy within a year, six within 5 years, and four after more than 5 years following diagnosis. Survivors had been on HAART for an average of 8.5 years with most taking three antiretrovirals once a day. The profile summary highlights the continued socioeconomic disadvantage this community endures.

**Table 1:** Demographic summary for survivors.

**Variables**	**Frequency (%)**
**Marital status**	
Single/separated	83.3%
Married	5.5%
Co-habiting	2.8%
Widowed	11.1%
**Housing**	
BC housing	27.7%
Temp hotel room	8.3%
Rent	55.5%
Shelter/street	11.1%
**Employment status**	
On disability allowance	47.2%
Seasonal/part-time job	27.8%
Unemployed	22.2%
Full time job	2.8%
**Education level**	
Some high school	44.4%
High school	27.7%
Elementary school	16.7%
University/college	11.1%
**Healthcare practitioner/service**	
Family doctor	88.9%
Emergency room	44%
Mental health counselor	38.8%
Eye specialist/optometrist	30.5%
Social worker	61.1%
Nurse	55.5%
Dietician	8.3%

We report on two broad categories (findings): (1) resilience as facilitator of HAART adherence; and (2) differential views on HT's impact. Resilience is expressed through nine concepts: use of Indigenous services, religion and spirituality; having cultural pride and engaging in mentorship; using one's personal effort; going through cognitive reflection to positive attitude/action; living in solitude or renunciation of group/family; having hopes and dreams, and reintegration; health literacy with knowledge acquisition; fear of sickness or death or a desire to live; and learned strictness and discipline. There were varied views on the impact of HT experienced with responses/expressions mediated by survivor status (see Table 2). Within the details of the quotes are references to nuanced but vital socioeconomic, environmental, and structural factors.

**Table 2:** Resiliency as facilitator of HAART adherence and differential views on HT impact.

**Categories**	**Concepts**
1) Resiliency as facilitator of HAART Adherence	Being resilient or achieving resilience through; • using Indigenous services, religion, and spirituality • having cultural pride, engaging in mentorship • using one's personal effort • going through cognitive reflection to positive attitude/action • living in solitude or group/family renunciation • having hopes and dreams, and reintegrating into society • health literacy with knowledge acquisition • fear of sickness or death, and a desire to live • learned strictness and discipline
2) Differential views on HT impact	• Survivor status determines response/expression • Response/expression is independent of survivor status

### Resiliency as Facilitator of HAART Adherence

The concepts presented were gleaned from survivor's responses in part to questions on their understanding of the term “resilience” and what they thought contributes to resilience with respect to their adherence to HAART.

Using direct word references and connotations of resilience (being “strong” or able to “overcome” are examples implied from subtext), survivors explained how they were resilient or achieved resilience and became HAART adherent.

#### Using Indigenous Services, Religion, and Spirituality

Participants mentioned their use of Indigenous services, the coming of one to understand his religion, and one's spirituality or belief on balance/harmony or a holistic way of life as fostering HAART adherence. Some commented;

“*Culture, traditional activities, that helps me” (A021)*.

“*If somebody has a balanced peace of mind, they go a long way” (A010)*.

“*I just believe that I have the power of Jesus that he wants me to survive. So, I gave up on being defeated and decided to have victory in my life” (A014)*.

#### Having Cultural Pride and Engaging in Mentorship

Having a sense of pride in one's culture and the role of mentoring others toward a better life helped some participants to adhere to medication. They explained;

“*I've got lots of pride in my heritage. That helps to take my medication” (A022 Indirect survivor)*.

“*I just want to survive and continue working and providing my services to Native Health as a survivor and encouragement to others to see that yes, I'm Indigenous, I'm a man with HIV, I can overcome it, with stride, have a better life. That we can carry on without defeat” (A014)*.

#### Using One's Personal Effort

Some participants attributed their HAART adherence to resilience through having a daily personal effort to take their medication. One commented;

“*Most of it is just from routine. I put myself in a routine and it's something that I don't say to myself well I'll take it later. It's my personal effort, my routine that I must do every day” (A011 Indirect survivor)*.

#### Going Through Cognitive Reflection to Positive Attitude/Action

Going through a process of cognitive reflection on the bad times, coupled with a positive attitude/desire to change into a better person and to take responsibility on one's life, led to personal development and ultimately HAART adherence. Some participants commented;

“*I used to get in trouble with the law before and I'd get picked up. I'd just start my medications right and then I'd be thrown in jail and then I'd miss completely. And, you think about it, then your resilience builds up. I take them every day. I never have a problem with that anymore” (A021)*.

“*I'm living a positive life today, resilient. Every day I try and live a positive life. I do positive things. I'll come here and do volunteering every day. Keep myself busy, that's how I take my medication” (A019)*.

“*When I was going to get high with drugs, I started developing a habit of going out and not coming home. I wouldn't take my meds so that's not a good thing. I know that when I'm clean and sober, that it helps me. I feel better about myself and now I'm back on track” (A025 Indirect survivor)*.

#### Living in Solitude or Renunciation of Group/Family

One's choice of living in solitude, helped a participant to be HAART adherent. Two commented;

“*I keep to myself. The less people I know, the better off I am” (A030)*.

“*Because of my family being like that. I slammed the door and I said you want me out of here, this is good. I'm leaving. I set my own positive thing, I left and never returned. I have a much better life, resilient life, now than I did” (A036)*.

#### Having Hopes and Dreams, and Reintegrating Into Society

Some survivors highlighted that their being adherent to HAART was due to their on-going growth in personal strength, relationships with others, appreciation of life, and recognition of new possibilities. They emphasized;

“*I want to get a job, get off the dope and I get back on track, get everything fixed, put some weight on and make something out of it before it ends too late or something like that” (A024 Indirect survivor)*.

“*To be productive in our world, other people that succeed leave a mark on you. I know lots of Native people that went to school, got a wonderful job…it gives me encouragement to keep going” (A010)*.

#### Health Literacy With Knowledge Acquisition

Better adherence to HAART was attributed to higher health literacy with greater acquisition of knowledge of medication management, consequences of unhealthy behavior, the development of resistance to medication and of the negative shifts in blood indices. Some participants commented;

“*My parents are not that well-educated, and it was worse, and the one after that, it started to clear up, like I'm doing the healing. So, I'm more resilient, yes” (A011 Indirect survivor)*.

“*Resistance to it and I won't be able to take the drug and it won't be effective. So, that's why it's always good to take it at the same time every day and taking it with food and being constant with it” (A024 Indirect survivor)*.

“*I take them every day. I mean I did at one time stop taking them and my numbers, like my viral load and CD4 was really bad and I know that when you don't take them how fast you get sick and it's very important to take them, but I stuck with it and now I don't have any problems” (A018)*.

#### Fear of Sickness or Death, and a Desire to Live

Most participants expressed a profound desire to live and/or fear of getting sick or dying as the main reason for their adherence to HAART. They explained;

“*I fear if I don't take it, I'll get sick from the virus. I don't want to get sick. I've been there, done that and I don't ever want to be like that again” (A011)*.

“*Seeing people with HIV really gives me insurance that if I don't take them, I might be on the way out. It gives me encouragement that I have friends and people around me, it just clarifies your mind and you set your goals to make sure you take your medication because you want to be alive” (A001 Indirect survivor)*.

#### Learned Strictness and Discipline

In response to how they maintained their adherence to HAART, a few participants emphasized that through the discipline and learned strictness of their parents, their experience of HT has indirectly cultivated in them a conviction to live as better persons. They explained;

“*My mom was strict, got the strictness from residential school, but she was a very good mother. What she learned from school; she never did to us kids. She got treated pretty badly in school. Yes, she told me about it. So, she always put an opposite spin toward us kids and this has helped” (A005 Indirect survivor)*.

“*Historic trauma and so forth, affected me ‘positively' somewhat. I guess my family were very strict and very religious. I've taken out of that. I've drawn from that and I feel that conviction to live on” (A007 Indirect survivor)*.

Other survivors explained how they would be made to fight other children in residential school to show strength or prove their worth in a group. One commented;

“*Attending residential school, I went through a lot of discipline. I grew up to take better care of myself and to help others when in trouble. I learnt to be strong in a fighting community. We were made to play war games, fighting physically. Split into teams and made to be friends again at supper time” (A036)*.

### Differential Views on HT's Impact

Many survivors reported having adopted negative coping strategies following HT, while others highlighted having developed adaptive coping strategies. Amid confounding factors, the extent to which they adopted health promoting behavior was mediated by whether the HT was experienced directly or indirectly.

#### Survivor Status Determines Response/Expression

A handful of participants believed that direct survivors of residential school are more affected by trauma, and their expression of it is greater than indirect survivors. They commented;

“*The next generations respond better to the trauma because they are more aware of what's going on and with the people that did that. It's not something they can take lightly” (A032)*.

“*I think residential school people would have more effect than ones who didn't go. Like my grandparents, theirs is really bad” (A011 Indirect survivor)*.

#### Response/Expression Is Independent of Survivor Status

A few participants expressed the view that individual responses and expressions of HT may vary irrespective of one's level of trauma experienced or survivor status. They commented;

“*Well, everybody gets a little bit of abuse out of it (residential school), right, there's no way out. But I don't feel like I've gone through too much damage” (A028Focus group)*.

“*Like I say, when things were happening to that person, they learned to block it out and thought of other things. Totally mentally, man. And you have damage and you learn to live with that though. Some people can hold their anger about it and some people can't” (A031Focus group)*.

“*The trauma is the same or may be different whether you went to residential school or not. Some people on reserves still got hit hard by their parents because they treated them the way they were treated in residential school” (A034)*.

## Discussion

Two broad categories (findings) emerged from this study. The first brings out summative expressions of resilience, showing how resilience promotes HAART adherence among the Indigenous men who participated in our study. These were: use of Indigenous services, religion and spirituality; having cultural pride and engaging in mentorship; use of one's personal effort; going through cognitive reflection to positive attitude/action; living a solitary life or renunciation of group/family when still engaged in self-destructive behavior; having hopes and dreams, and reintegration into society; health literacy with knowledge acquisition; fear of sickness or death, and a desire to live; and learned strictness and discipline. The second category (finding) suggested varied views on the impact of HT experienced with responses/expressions mediated by survivor status.

Our study found many factors linked with previous research indicating health maintaining behavior promotes healing from HT in Indigenous peoples (33, 49). The study shows that, for Indigenous men, not only does resilience come from protective factors, such as cognitive reflection, a positive attitude, personal effort, living in solitude or renunciation of group/family, but also from knowledge about HT and its effects. Individuals also showed health promoting behaviors despite residing in communities or environments that marginalize, discriminate against, or heavily stigmatize those living with HIV. The references by survivors to the influence of relationships, employment or volunteer work, service access or availability, as well as social circumstances (differential exposure to cycles of trauma, stigma) illustrate this. These intertwining factors need therefore be considered as they determine men's abilities to adopt protective strategies. In line with evidence that the experience and manifest response to trauma may differ (17, 50), our study adds evidence that the HT complex includes health behaviors mediated by the experience of HT across survivor status levels.

### Minding Your Language: Intergenerational Historic Trauma Is Detrimental

The experience of extensive repetitive trauma, and the notions of “gaining a sense of accomplishment through being coerced into fighting” others in residential school, for some survivors, expresses resistance and adaptation to oppressive environments. In some Indigenous cultures, this may reflect acceptance that this was the Creator's gift or challenge, whether untenable, detrimental or beneficial, for them to survive (51, 52). That they have indirectly learned to be a better person and developed a conviction to live does not reflect “positive effect” in a literal sense, because abuse in all its forms is never beneficial. What can be positive is the person's response to abuse, as exemplified by our study participants.

We call for experiential stories of success and healing, in the face of continued injustices and structural influences that deny Indigeneity, to be celebrated, the persisting reality of colonization calls for mindful reflection on the use of terms that may continue to suppress, stereotype and fuel negative assumptions about Indigenous peoples. Being able to adhere to medication and continuing along the healing journey should not only be secondary to mitigation of larger ongoing socio-economic, environmental, and structural factors but should also be seen with due reflection as a liberating praxis. The impetus in clinical care must therefore be to view health promoting behavior broadly, and to foster it in a culturally safe manner (25).

### Differential Responses and Their Place in the Healing Journey

As indicated in previous research (49), the wide range of responses to HT can have devastating effects on subsequent generations. However, predicting that HT affects individuals in equal measure is unfounded because the trauma faced, expression exhibited and health promoting resources and behavior built by each survivor is complex, and is influenced by many factors. Furthermore, the fact that an individual or group may positively adapt today may not be a shield against future risk of developing dysfunctional behavior as the survivors continue to experience trauma, including in a vicarious nature (53), as subsequent generations share in the pain from the collective memory of direct survivors, and the loss of oral traditions and storytelling.

Although storytelling seldom keeps some wounds open, in order to heal and attain full reconciliation, one must, paradoxically, first remember in order to forget (54). Survivors' stories must continue to be told as they not only foster the healing process but also allow for skeptics alike to bear witness, and inspire those in need of healing. The healing process must co-exist with cultivation of the ability, on the part of decision makers and care providers, to empathize and practice cultural humility and vigilance toward promoting healthy lives (55).

In the biopsychosocial sphere, this unequal association between dysfunctional behavior development and adaptation is supported by a demonstrated bidirectional link between functioning, levels of stress hormones and factors that contribute to resilience and stress outcomes (28, 56). Studies have shown that resilience is fostered by adoption of personal control, positive affect, optimism, and social support. These factors help protect against the deleterious influences of stressors on physiology in general. The reverse is true with resilience as there is evidence that it is influenced by immune mediators. These mediate the way the brain processes information and how one responds physiologically, emotionally and behaviorally (57, 58). The experience of HT may therefore not guarantee development of health promoting behavior in all survivors, neither will the trauma always result in dysphoric or psychopathological dysfunction.

### The Balance of Solitude and Support Through Parenting Style

The finding that HAART adherence may be attributed to living in solitude, in part secondary to HT, highlights the often neglected but important contribution of solitude toward emotional maturity (59). Seeking fulfillment in a life of solitude should not be treated as necessarily pathological since an avoidant person may rightly shy away from behavioral disorganization to find meaning and order in life (60). Further, having family, friends, or group support may not be ideal because for some of the men, living in communities where services and supports are limited, one may be more likely to succumb to negative peer pressure to engage in unhealthy behaviors to reduce their HT pain – given the relative absence of positive options (61, 62).

The relation of parents to their children is not only of crucial importance for their internalization of values and beliefs but also a consistent predictor of positive childhood and adolescent outcomes as it influences their behaviors (63). Survivors' attribution of their building of resilience to parenting styles may mean that their parents, having experienced strictness themselves (in the negative punitive sense), adopted an authoritative and religious parenting style of gentle and positive discipline (studious teaching), that conforms to a supportive, less-controlling non-punitive model (64). These findings are in line with prior research and theory that shows that such parenting promotes emerging moral awareness, commitment to goal accomplishments, the ability to make better choices, accountability, good behavior, self-esteem and confidence-building, resilience, healthy development, and well-being (64, 65). The parental religiosity highlighted in this study may not only complement these attributes but also offers grounding of the moral beliefs and healthier behavioral norms in an ideological, transcendent worldview that gives meaning and continually shapes behaviors learnt.

Of note, for minority communities residing in high-risk environments, including of these Indigenous men, parental strictness may be more likely interpreted as necessary and acceptable, in essence normative, and an important safety skills-building and socialization mechanism that yields positive effects on adolescent outcomes such as being less prone to externalizing behaviors or less likely to engage in illicit drug use (65).

### Knowledge Acquisition and the Interplay With Survivor Status

Positive psychology generated from acquisition of medical knowledge potentially promotes resilience and HAART adherence, decreases the viral load and slows down CD4 + cell count decline rates in those with a trauma history (28, 66). In this study, knowing the dire consequences of non-adherence (negative shifts in blood indices and development of resistance) encouraged health promoting behavior and fostered HAART adherence.

The impetus to use available health information, be resilient, and adhere to HAART may be mediated by not only education but also one's survivor status, coupled with ongoing influence of resources on one's health in adulthood (e.g., depending on employment status). Some survivors may be more receptive to and proactive in seeking health promoting messages or services as they are influenced by knowledge and skills gained through education.

Furthermore, needs may be different for those who perceive themselves as less affected by HT, perhaps the more informed indirect survivors. Navigating the daily struggle to harness resources and work toward achieving a better future may hold more importance than pondering on past adversity (29). For some, resilience may thus be reflected through achieving balance in the facets of health and well-being, which include one's environment, when needs are seen as interdependent in nature (67).

### Commitment to Cultural Practices, Religious, and Spiritual Reverence

Previous studies show that religious and spiritual reverence foster HAART adherence and healing (68, 69). This was true for survivors in this study who attributed their health promoting behavior to inner strengths and purpose for living. Such piety is a resource for resilience and is closely bound with culture and ways of living in Indigenous communities (70, 71). Many extend their positive attitude, expressing self through cultural practices and language, to having cultural pride that helps them from courting chaos or undesirable marginalization, and generating meaning of one's HIV situation (28). They believe and hope again, and confidently live without fear of sickness or death, while maintaining HAART adherence. This cultural pride affords them strength and resilience culminating in a new level of posttraumatic growth as they appreciate life and the possibility of reintegration through new opportunities (72, 73). Posttraumatic growth is based on the assumption that many people, through cognitive effort, go through positive psychological change, become hopeful, find meaning in their trauma, grow and gain satisfaction in life (29, 74).

Development of health promoting behavior in Indigenous men living with HIV, as well as their communities, can thus promote the long process of healing that allows reclaiming cultural identity, the rejuvenation of rituals/practices, beliefs, and institutions retaining key elements that preserve the distinctness of Indigenous communities and systems.

### Implications of the Study Findings for Policy and Practice

Given how health promoting factors operate in a marginalized community such as that of these HT survivors, implementing effective interventions/services may often require:

- mitigation of the effects of HT by providing culturally safe services for these Indigenous men through health care providers who strive to be non-prejudicial but culturally connected, embracing cultural safety, and bringing together their health literacy with cultural humility (55). This can be done by first assessing individual survivor's unique circumstances, i.e., survivor status, trauma genesis, life journey, current environment, individual needs, personal attributes, and readiness for and acceptance of HAART to foster the positive behavioral process.
- allowing for prosperity in Indigenous men through promotion of tolerance, cultural diversity, and development of one's own preferred spiritual and/religious belief. Appropriate resources for this should be made more accessible in all organizations offering healthcare services for those living with HIV and for survivors of trauma.
- more attention and sensitivity to the promotion of use of words such as “positive effect” when communicating a survivor's response to HT as such words may be either limited, convey the opposite meaning, or not emancipatory to Indigenous peoples. Indigenous peoples may find themselves constrained to tell their stories and translate their concerns into other languages in a public space where Indigenous voices are quashed into silence (75).
- application of positive reinforcement and motivational interviewing with feedback about confirmable favorable shifts in blood indices discussed during clinic visits to promote HAART adherence and resilience.
- the need to accommodate of both ends of the spectrum of promoting support through appropriate parenting style and one's choice of solitude as both can be therapeutic and may foster resilience and better adherence to HAART for survivors.
- capacity building and job placements in Indigenous communities to effectively advance health promoting behaviors.
- attention to food security as this helps mitigate the risk of developing side effects and/or resistance to medications thereby promoting adherence.

## Implications of the Study Findings for Future Research

Study findings raise a few areas for future research. These include: development of an evidence base of cost-benefit culturally safe measures and transferable competencies for evaluation of how spiritual healthcare and Indigenous healing practices are applied in communities. This would help support Indigenous men and their communities' preferences for appropriate care during their healing journey; an enquiry into the role of positive psychology in Indigenous men on improvements of laboratory indices (viral load and CD4 + cell counts) for monitoring HAART adherence and response to therapy; understanding how expressions of intergenerational HT and resilience in youth and women are mediated by survivor status; an exploration of how those residing on-reserve view Indigenous resilience with respect to its role in mitigation of unhealthy behaviors and promotion healing from HT; and, an enquiry to better understand how different parenting styles and religiosity in Indigenous communities may influence the building of health-promoting behavior and resilience. Such evidence may shed light into what relevant skills to promote in parents for attainment of better health and well-being in Indigenous communities.

## Limitations

Policy makers, healthcare providers and researchers may not be able to generalize the study results to all Indigenous groups or contexts as participants were mostly from the city of greater Vancouver. It was not possible to schedule interviews with some participants as they exercised their autonomy and declined consent seeing themselves as not having been HAART adherent at the time. Bias could have thus existed in the type of individual who consented to participate. This might result in disproportionately highlighting the experience of survivors who have adopted health promoting behaviors. Further, the intergenerational and survivor status comparison on the experiences and expressions of HT was limited following challenges including inability to access medical records and the fact that many youths remain undiagnosed. However, to help in possible transferability efforts or decisions to other contexts/ population groups where applicable, we included sufficient quotes, and the assumptions made were recorded.

## Conclusions

Most Indigenous men in this study demonstrate health promoting behavior, stay on HAART and have better health and well-being even if the environments they live in are marginalized or heavily stigmatizing. This study contributes to the field of HIV/AIDS research by showing that resilience in these men comes from protective factors that include cognitive reflection, a positive attitude, personal effort, and solitude or renunciation of group/family. The varied responses noted on HT magnitude and complexity of experiences, expressions and impacts across survivor status in these Indigenous men also add to available literature. Light is shed into different approaches to managing HT, considering the confounding effects of resource inadequacy, disobliging environmental conditions, and varied survivor attributes and individual life journeys. With a consideration of areas of strength and adaptation, this study offers implications for research and recommendations to improve treatment-adherent behavior, fostering healing from HT, and reducing deaths due to HIV/AIDS.

## Data Availability Statement

All datasets generated for this study are included in the article/supplementary material.

## Ethics Statement

The studies involving human participants were reviewed and approved by 1. University of Northern British Columbia 2. Vancouver Native Health Society Research Committee. The patients/participants provided their written informed consent to participate in this study.

## Author Contributions

MC conducted the interviews and focus groups. MC and JL were involved in the project's stages of engagement, conceptualization, design, analysis, and manuscript write up. JM, NC, and HH contributed their specialty knowledge in data analysis and manuscript write up. RC reviewed and provided comments for manuscript editing. All authors contributed to and have approved the final manuscript.

## Conflict of Interest

The authors declare that the research was conducted in the absence of any commercial or financial relationships that could be construed as a potential conflict of interest.
